# Clinical neurophysiology of migraine with aura

**DOI:** 10.1186/s10194-019-0997-9

**Published:** 2019-04-29

**Authors:** Gianluca Coppola, Cherubino Di Lorenzo, Vincenzo Parisi, Marco Lisicki, Mariano Serrao, Francesco Pierelli

**Affiliations:** 1 Department of Medico-Surgical Sciences and Biotechnologies, Sapienza University of Rome Polo Pontino, Corso della Repubblica, 79–04100 Latina, Italy; 2 IRCCS – Don Gnocchi, Piazzale Morandi, 6–20121 Milan, Italy; 3 IRCCS – Fondazione Bietti, via livenza, 3–00198 Rome, Italy; 4 Headache Research Unit, University of Liège, Department of Neurology-Citadelle Hospital, Boulevard du Douzième de Ligne, 1-400 Liège, Belgium; 5 IRCCS – Neuromed, Via Atinense, 18-86077 Pozzilli, (IS) Italy

**Keywords:** Cortical excitability, Habituation, Slow rhythms, Interhemispheric asymmetry, Neuromodulation, Paradoxical responses

## Abstract

**Background:**

The purpose of this review is to provide a comprehensive overview of the findings of clinical electrophysiology studies aimed to investigate changes in information processing of migraine with aura patients.

**Main body:**

Abnormalities in alpha rhythm power and symmetry, the presence of slowing, and increased information flow in a wide range of frequency bands often characterize the spontaneous EEG activity of MA. Higher grand-average cortical response amplitudes, an increased interhemispheric response asymmetry, and lack of amplitude habituation were less consistently demonstrated in response to any kind of sensory stimulation in MA patients. Studies with single-pulse and repetitive transcranial magnetic stimulation (TMS) have reported abnormal cortical responsivity manifesting as greater motor evoked potential (MEP) amplitude, lower threshold for phosphenes production, and paradoxical effects in response to both depressing or enhancing repetitive TMS methodologies. Studies of the trigeminal system in MA are sparse and the few available showed lack of blink reflex habituation and abnormal findings on SFEMG reflecting subclinical, probably inherited, dysfunctions of neuromuscular transmission. The limited studies that were able to investigate patients during the aura revealed suppression of evoked potentials, desynchronization in extrastriate areas and in the temporal lobe, and large variations in direct current potentials with magnetoelectroencephalography. Contrary to what has been observed in the most common forms of migraine, patients with familial hemiplegic migraine show greater habituation in response to visual and trigeminal stimuli, as well as a higher motor threshold and a lower MEP amplitude than healthy subjects.

**Conclusion:**

Since most of the electrophysiological abnormalities mentioned above were more frequently present and had a greater amplitude in migraine with aura than in migraine without aura, neurophysiological techniques have been shown to be of great help in the search for the pathophysiological basis of migraine aura.

## Introduction

During the last 50 years, researchers dedicated their projects to the understanding of neurophysiological peculiarities of the migraine brain which might predispose to the recurrence of migraine attacks. This implies that most of the possible electrophysiological signatures of these subtle underlying factors were detected between migraine attacks, fluctuating depending on the distance from the last or the next attack. Moreover, even though amongst the migraineurs those who experience aura (MA) exhibit more pronounced clinical manifestations, these patients have been less frequently studied from a neurophysiological point of view. This is, by the way, due to its lower prevalence in comparison to the commonest migraine without aura (MO) and because of the short duration of the aura phase. In fact, focal neurological symptoms which precede or accompany the headache phase (when presents), last no more than 60 min with visual - the most common aura symptom - followed by sensory and aphasic auras [1, 2]. However, a significant proportion of auras may last longer than one hour and may configure the diagnosis of persistent aura without infarction [3].

The electrocortical phenomenon of cortical spreading depression (CSD) has been implicated in the genesis of migraine aura: it is a wave of neuronal hyperactivity followed by a wave of hypoactivity which often spreads postero-anteriorly and can reach the parietal and/or temporal lobes travelling at a speed of approx. 3 mm/min [4]. After the first description of CSD in animals by Leão [5] up-till-now only indirect evidence for CSD in migraine patients derived from functional MRI [6–8] and magnetoencephalographic [9, 10] studies has been gathered. Although in animal models CSD is able to ignite the trigeminovascular system, which is the condition for a headache to start, less is known about the possible biomarkers of CSD during the interictal migraine that might predispose to the aura and, perhaps, to the attack itself.

To better understand aura-related changes in sensory processing, several independent research groups have dedicated to the study of electrocortical signals during different phases of the migraine cycle using different sensory stimuli, or single or repetitive neuromodulatory techniques delivered over the scalp. Interestingly, none of the published studies assessed patients suffering exclusively from migraine with aura, at least with respect to the most common episodic forms of migraine. This has happened not only because patients suffering exclusively from migraine attacks preceded by aura are difficult to find, but also because for many authors the two conditions of MO and MA are variable clinical manifestations of substantially the same genetic disorder [11].The purpose of this review is to provide a comprehensive overview of the findings of clinical electrophysiology studies aimed to investigate changes in sensory processing of migraine with aura patients.

### Data overview

#### Electroencephalography (EEG)

Several decades passed since the pioneering electroencephalographic studies emphasizing abnormal electrocortical activities in migraine [12]. During the last 60 years of publication, the most frequently described electrocortical phenomena in migraine patients were the so-called H response to flicker stimulation – also known as enhanced photic driving (PD) –, and the abnormal resting-state EEG rhythmic activity.

Enhanced PD of EEG during intermittent photic stimulation using fast Fourier transform analysis on steady-state visual evoked potentials (SS-VEPs), the so-called H response, was more prevalent in migraine patients than in healthy controls. Researchers observed that the fundamental components of the EEG spectra were increased equally in both MA and MO [13, 14], predominantly in the temporo-parietal regions, with reduced interhemispheric coherence in fronto-temporo-parietal areas [13]. The same phenomenon tends to be present also in juvenile MA patients [14]. H-response showed a sensitivity of 86.4% and a specificity of 97.5% in MA and MO patients, but not in patients affected from basilar migraine [15]. De Tommaso and coworkers [16] observed that, although in both MO and MA groups PD was significantly enhanced with respect to controls, those patients experiencing aura showed more pronounced decreased phase synchronization between beta rhythms and higher Granger causality values – measuring the flow of connections and information across different brain areas – during light stimulation compared to MO patients. Response to photic stimulation was less represented in MA than in MO patients in two studies [17, 18].

During the interictal period of MA patients, quantitative analysis of *spontaneous electroencephalographic activity* showed alpha rhythm and peak frequency asymmetries over the posterior regions, increased power of alpha rhythm [19], and widespread increase in delta [14] and theta [14, 19] total power in comparison with healthy controls. Reduction of alpha rhythm [20] or unilateral reduction of alpha and theta activity was detected in MA patients with a pure visual aura [21], mostly contralateral to the neurological signs [21]. MA patients had greater alpha peak power interhemispheric asymmetry, chiefly in the posterior regions, and unrelated to the headache side, than MO [14, 22]. In a resting state effective neural connectivity EEG study, MA patients showed higher flow of information transfer in beta band compared to MO patients and controls [23]. When using a checkerboard pattern for visual stimulation, MA patients showed increased transfer entropy with high density of information flow in the frontal regions in all the bands of rhythmic activity as compared to MO patients [23]. Using magnetoencephalography (MEG), researchers found that MA patients had significantly increased functional connectivity in the theta (4–8 Hz) band in the occipital area as compared with patients not experiencing aura [24]. It is interesting to note that functional connectivity anomalies at the level of the frontal and occipital networks were detected also with the method of resting-state functional MRI [25–27].

In summary, resting electric and magnetic activity may help to better differentiate MA from MO patients than PD.

#### Evoked potentials

With the help of cortical evoked potentials, higher cortical response amplitudes, an increased interhemispheric response asymmetry, and a deficit of response amplitude decrement were demonstrated by using different types of sensory stimuli and techniques in most of the MA patients.

#### Grand-average EP amplitude

Because in most cases the aura is visual, most of the published studies investigated visual evoked potentials (VEPs) to search for cerebral signatures associated with migraine aura. By analysing the evoked responses in a classical way of averaging a large quantity of trials, mainly increased amplitudes of steady-state (SS) or transient VEPs have been discovered in MA patients during attack-free intervals.

In some reports the grand-average of VEP N75-P100 and/or P100-N145 amplitudes has been found greater in MA patients than in controls [28–33] and/or in MO patients [28, 34, 35]. The amplitude of SS-VEP harmonics was also higher in MA than in MO or controls [36]. In other studies, on the contrary, VEP amplitudes were found reduced in MA [37], even when compared to MO [38]. Most often, VEP amplitudes in MA were reported to be in the normal range [39–45].

Decreased amplitude of the prerolandic component (N20) of somatosensory evoked potentials (SSEPs) in both MO and MA patients has been found in one study [46], but amplitudes were within the normal range in others [47–49].

Most of the researchers who recorded short-latency brainstem auditory evoked potentials (BAEP) were not able to find any interictal abnormalities in migraine, probably because they pooled patients with different migraine phenotypes (MO and MA or different MA subtypes) in different proportions in a single group (see Table 5 in [50]). Higher P300 event-related potentials (ERPs) is a common finding in MA compared with other types of primary headaches [51, 52]. In comparison with controls, basic P300 amplitude tended to be greater in a mixed group of MO and MA patients. Moreover, P300 amplitude was significantly reduced during mind wandering relative to on-task periods in migraineurs, contrasting to what happened in healthy controls. Authors argued that a more consistent propensity towards engaging in response attenuation during mind wandering states may provide migraineurs with an alternative compensatory strategy for reducing stimulus overload in cortex [53].

To sum up, using EPs and ERPs, researchers found that the frequently reported increase in grand-average neural response to any kind of sensory stimuli in MA group is conceivably due to deficient short-term and long-term adaptive processes to external stimuli.

#### Interhemispheric asymmetry

Asymmetric neural activities in steady-state VEP amplitude, transient VEP P100 amplitude distribution and in N70 components were detected by some, both related [29, 54, 55] or not [56–58] with side of visual aura. A significant interhemispheric asymmetry of the N30 component amplitude has been observed in the MA group in comparison with control subjects [46].

Similar to the results of VEP and SSEP studies, in one study mean interhemispheric asymmetries of all BAEP peak latencies (except peak IV and VI) were significantly increased in MO and MA patients as compared to those of the control group, despite the fact that the MA group included hemiplegic, and brainstem migraine [59]. This datum was not confirmed in a more recent study [60].

#### Response habituation

Analysing discrete blocks of small amounts of traces, authors found that during repetitive and stereotyped stimulus presentation, VEP amplitudes augmented progressively instead of diminishing (i.e. they lacked habituation) equally in MO and, sometimes even more so, in MA patients between attacks [39–42, 44, 45, 61–63]. Some studies failed to confirm deficit of amplitude habituation in migraineurs during the interictal period [30, 43, 64, 65]. Deficient lateral inhibitory mechanisms within the visual cortex might be one of the culprits for this abnormal information processing in migraine as clearly showed with SS-VEPs elicited by a windmill-dartboard pattern [41]. Defective inhibitory mechanisms within the visual cortex in MA, but not in MO, were further confirmed in a paired-pulse flash-VEPs study [66].

Since in MA patients, different aura phenotypes may be underpinned by different pathophysiological mechanisms, we studied VEP amplitude and habituation in a subgroup of MA with exclusively visual auras and another with visual aura followed by somatosensory and/or dysphasic complex neurological auras [67]. We found a significant sustained increase of VEP amplitude in MA with complex aura – interpreted as a genuine increase in cortical excitability –, while it was within the normal range in migraine with pure visual aura. In both subgroups VEP habituation was equally deficient as compared with healthy controls, yet in those patients with complex aura the more pronounced the VEP habituation deficit the longer the distance from the last migraine attack [67], as previously observed in another study from the same research group, but in a mixed group of MO and MA [41]. In a study where VEPs were co-recorded with MRI spectroscopy, MA patients showed greater VEP amplitude and lack of habituation as compared with healthy controls [68]. More interestingly, both cortical excitability enhancing and inhibiting transcranial direct current stimulation procedures were unable to induce significant changes in VEP amplitudes in MA, while they significantly potentiated and diminished N1-P1 VEP amplitude in healthy controls keeping a correlation with glutamate signals [68].

In accordance with VEP studies, a significant habituation deficit has been detected interictally in MA recording SSEPs [69] and auditory evoked potentials (AEPs) [70]. Lack of response habituation is also responsible for the strong interictal dependence of AEPs on stimulus intensity, that, in turn, is known to be inversely related to cerebral serotonergic transmission [44, 70]. There is also evidence for a loss of habituation during cognitive potentials as assessed by recording P300 amplitude in MA [51, 52].

#### Techniques of neuromodulation

Studies with single-pulse and repetitive transcranial magnetic stimulation (TMS) have reported abnormal cortical responsivity revealed as greater motor evoked potential (MEP) amplitude, lower threshold for phosphenes production, and paradoxical effects in response to both depressing or enhancing repetitive TMS (rTMS) methodologies, predominantly in migraine with aura. Magneto-phosphenes measurements of MA patients were significantly lower - revealing higher excitability levels - than healthy controls measurements in most of [71–77], but not in all [78–82], the studies. Naeije et al. [83] successfully used TMS in discriminating transient ischemic attacks of vascular origin from migraine aura without headache. Greater motor-evoked potential amplitude in response to increasing intensity of stimuli in MA patients compared to controls, with its normalization after levetiracetam preventive treatment, was revealed in one study [84]. A group of authors observed that inhibitory trains of rTMS delivered over the motor cortex of MA significantly activates rather than inhibiting intracortical facilitatory circuits, which might depend on glutamatergic synaptic mechanisms [85]. A datum further confirmed delivering inhibitory rTMS over V1 and assessing phosphene threshold which was normally enhanced in controls, but reduced in MA [80], and raised again after prophylactic treatment with valproate [86]. Nonetheless, other studies provided evidence for the same paradoxical effects over M1 since facilitatory rTMS recruited the excitatory circuits in mechanisms of glutamate-dependent short-term synaptic potentiation more easily in MA patients than in those without and healthy controls [87, 88]. On the other hand, excitatory 5 Hz-rTMS at 130% of the resting motor threshold over M1 determines a significant depression in MEP size in MA rather than a clear MEP facilitation as in healthy subjects [87].

In sum, both the paradoxical rTMS response and habituation deficit point to altered synaptic plasticity mechanisms, which prevent the immediate and longer-lasting cortical changes that reflect adaptation to repeated stimulations, i.e. learning and memory. Further studies are needed to verify whether these aberrant ways of responding of the cortex to neuromodulation are related to abnormal thalamic control [89] or to a failure of the hypothalamic functional connectivity as recently described in a single MA patient with resting-state MRI [90].

#### Electromyographic techniques

Even though brainstem trigeminal nuclei are well knowingly deeply involved in the pathophysiology of migraine without aura, the studies of the trigeminal system in MA are still sparse.

Perrotta et al. [91] studied a group of MA patients between attacks by measuring the bilateral polysynaptic R2 component of the nociceptive blink reflex (nBR). They found comparable normal baseline activation to noxious supraorbital stimulation with delayed response lack of habituation in both MO and MA as compared with controls. However, they noted that despite the habituation deficit was equally present in both migraine groups, that of MA tended to be less pronounced than that observed in MO. Moreover, in the MA group the higher the frequency of the migraine attacks the more pronounced the habituation of the nBR R2 component [91]. The same correlation was previously observed also in a group of MO patients [92], and might be explained by the fact that patients with high attack frequency are more likely to be recorded in a closer temporal relationship to an attack, when nBR habituation tends to normalize [93].

With the scope to correlate interictal neurophysiological abnormalities of migraine, especially with aura, with a specific genotype, researchers recorded single-fibre electromyography (SFEMG) to explore neuromuscular transmission, as a surrogate biomarker of presynaptic P/Q Ca2+ channels function, in a wide range of migraine aura subtypes. Abnormal finding on SFEMG reflecting subclinical disfunctions of neuromuscular transmission have been detected in patients suffering from MA in between attacks. Patients with unilateral sensorimotor symptoms and/or visual scotoma, other aura symptoms such as sensory/motor disturbances, and/or aphasia, and/or vertigo had noticeable abnormal SFEMG [94, 95]. These findings were confirmed in a larger group of MA patients where subclinical abnormalities of neuromuscular transmission were progressively more noticeable starting from patients with mixed MO and MA to migraine with prolonged aura, with migraine with typical aura falling in between the two [96, 97].

In one pilot study, the mild single endplate abnormalities detected by SFEMG in 3 MA patients disappeared during acetazolamide treatment in parallel with clinical improvement [98].

### Neurophysiological findings during migraine aura

So far, few studies during the transient phase of migraine aura have been performed.

During visual aura and/or early headache phase, either mild asymmetry of slow waves in the fronto-temporo-occipital areas contralateral to the visual field defect disappearing during the headache phase [99–101] or normal [100] EEG recording have been reported. In some patients, identical abnormal slow waves were present interictally [101]. In a patient who underwent spectral analysis and topographic EEG mapping during complex aura, posterior-anterior spreading of slow activities and depression of alpha activity contralateral to the neurological signs were the prominent findings [21].

In a MA patient spontaneously experiencing a scintillating scotoma in the right hemifield, MEG recording showed alpha rhythm event-related desynchronization in the contralateral extra-striate and temporal cortex for the duration of the focal visual symptoms, and gamma band desynchronization peaking 10 min following the aura [10]. In another MEG study, slow direct current potential shifts - very similar to those found during CSD in animals [102] and abnormal spread of visual-evoked activity have been observed during the occurrence of spontaneous and visually induced migraine aura [9].

During the visual aura the hemisphere contralateral to the field defect showed suppression or complete abolition of the first three components of the flash VEPs [103] and of the parietal component of the SSEPs [104]. The latter component showed also delayed latency and increase central conduction time [104]. All the abnormal neurophysiological parameters gradually returned to normal during the subsequent headache phase [103, 104].

Chen and colleagues [62] showed that a group of 6 patients affected from persistent aura (PA) without infarction tended to have an early and more intense P100 MEG response to checkerboard pattern reversal than MO, MA, ictal migraineurs, and chronic migraine. Moreover, compared to the interictal MO and MA groups, the patients with PA showed the more pronounced lack of P100m habituation during stimulus repetition [62].

### Neurophysiological findings in other non-common auras

Electroencephalographic abnormalities during acute attacks of hemiplegic migraine are often described. During long-lasting attacks of hemiplegic migraine, unilateral or bilateral delta EEG activity – sometimes spreading postero-anteriorly [105] – and reduction of alpha are often recorded [106–115], while theta abnormalities have been described during the interictal phase [109, 116].

Adult and adolescent patients with brainstem aura (previously termed basilar type migraine) with disturbed consciousness may have severe clinically relevant EEG-slowing or generalized spike and wave complexes that may last for several days [117–127]. Hansen and colleagues measured habituation of VEPs, IDAP, and nBR in a group of nine genotyped familiar hemiplegic migraine (FHM) patients (FHM-1 *N* = 5; FHM-2 *N* = 4) and in a group of seven healthy controls [128]. Contrary to the commonest forms of episodic MO and MA, patients with FHM had significantly more pronounced habituation during VEPs and nBR recordings than controls, with no differences during IDAP, despite a tendency for the slope to be steeper in the patients group [128].

In one study, a group of ten patients with FHM showed basically higher resting motor threshold, longer central conduction time, and lower MEP amplitude on the ictally paretic side than on the non-affected side, while MEP amplitude were significantly increased in a group of MA [129].

## Discussion

There is no common agreement yet about what causes and where the cascade of events that lead to the focal neurological symptoms of migraine aura starts and, in most but not all the cases, about its link to the headache phase. However, experimental evidences point towards sequential activation of first-order or second-order trigeminovascular nociceptors via CSD waves [130]. More likely, a cyclical recurrent malfunction of the pain modulatory structures located at the brainstem level (raphe magnus, locus coeruleus and other aminergic nuclei) could play a major role in determining the start of the cascade of events that leads, on the one hand, to the beginning of CSD, on the other hand to the onset of pain [131, 132]. Several evidences point toward an involvement of brainstem both in MO and MA pathogenesis. An hyperperfusion within the brainstem during migraine aura was seen in one study [133], the same area that has already been reported to be implicated in the generation of attacks in groups of MO [134, 135] or mixed MO and MA [136, 137]. Moreover, with the brainstem, authors found abnormal macrostructure and functional activation of widespread subcortico/cortical areas, such as neurolimbic area [138], periaqueductal grey matter [139], hypothalamus [90], thalamus [140], trigemino-thalamic tract [139], visual [133, 141] and somatosensory [142] cortex. The involvement of such a wide variety of brain structures in MA has already been witnessed many times and long before by the neurophysiological studies reviewed here. The results can be summarized as follows (see also Table 1):

- Quantitative EEG rather consistently reported enhanced interictal photic driving, so called “H-response”, as well as excess of slow and hyper synchronized alpha rhythmic activity.
- Less consistently, EP and ERP studies showed many cases of cortical hyper-reactivity to sensory stimuli, including cognitive ones. When present, this heightened cortical response in MA was even more pronounced than in MO.
- Both lack of sensory habituation, of cortical inhibition, and paradoxical responses obtained after non-invasive brain neuromodulation, such as increased or decreased responses respectively to inhibiting or activating TMS, are commonly observed in MA. Like in MO, interictal abnormal cortical information processing in MA may depends on the time evolved since the last attack.
- Because the aura has numerous and varied clinical features, it may not be a single entity, but correspond to a spectrum of clinical subtypes that probably differ from a pathophysiological point of view. In fact, neurophysiological patterns distinguish between patients who experience pure visual auras from those with prolonged, somatosensory, dysphasic or motor, auras.
- Few researchers were able indeed to study patients during an aura. From a functional point of view, they detected unilateral disturbances of cortical electrogenesis – which might reflect an underlying metabolic abnormality [143] –, desynchronized visual and somatosensory potentials, signal desynchronization in extrastriate and temporal regions with MEG and large variations in direct current potentials, much like those seen during CSD in animal models.
- Few reports in FHM support the concept that various pathophysiological aspects differ between FHM and MO/MA, including cortical and brain stem responsiveness.

**Table 1:** Synoptic table of neurophysiological changes comparing episodic migraine with aura (MA) between attacks, during the aura phase, and familiar hemiplegic migraine (FHM). Arrows indicate the direction of change

	Episodic migraine with aura between attacks	Episodic migraine during the aura	FHM
Technique			
EEG & MEG	↑ photic driving, ↑ of slow, and hyper synchronized alpha rhythmic activity	On EEG, mild asymmetry of slow waves contralateral to the visual fiend defect	Unilateral or bilateral delta EEG activity – sometimes spreading postero-anteriorly – and ↓ of alpha
		On MEG, alpha and gamma rhythm ERD contralateral to the visual fiend defect, slow direct current potential shifts	
EP & ERP grand-average	↑ amplitude (more than in MO) or normal, ↓ thalamocortical activity	↓ or abolition of cortical EPs in the hemisphere contralateral to the field defect	
EP & ERP Habituation	↓ habituation (more than in MO) or normal	↓ habituation during persistent aura without infarction	Significantly more pronounced habituation during VEPs and nBR recordings than controls
TMS	Paradoxical effects to enhancing and reducing paradigms of stimulation		↓ MEP amplitude on the ictally paretic side
EMG recordings	↓ Habituation of nBR, subclinical abnormalities of neuromuscular transmission on SFEMG		

*EEG* electroencephalography, *EMG* electromyography, *EP*, evoked potentials (visual, somatosensory and auditory), *ERD* event-related desynchronization, *ERP* event-related potentials, *MEG* magnetoelectroencephalography, *MEP* motor evoked potential, *MO* episodic migraine without aura, *nBR* nociceptive blinck reflex, *TMS* transcranial magnetic stimulation

We hypothesized that the neurophysiological pattern which characterizes MA patients of an abnormal cortical rhythmic activity, an increased cortical responsivity, and deficient lateral inhibition may be ascribed to a “thalamo-cortical dysrhythmia” (TCD) [40], that is a theory used to explain numerous functional brain disorders [144]. The TCD theory assumes that, in presence of a functional disconnection of the thalamus from subcortical areas (as for instance the brainstem monoaminergic nuclei) a change of rhythmic thalamocortical activity may occur favouring low frequency activity at the cortical level. This will consequently reduce firing rates of excitatory pyramidal cells at the beginning, and of fast-spiking inhibitory interneurons during stimulus repetition [145]. In support of this theoretical explanation, some authors found a tendency to a reduction [48] or a full reduction of the amplitude of the pre-synaptic burst of high-frequency oscillatory activity embedded in the common SSEPs reflecting thalamocortical activity [47] in MA patients between attacks. In another study, a rise of the early high-frequency oscillatory (HFOs) activity embedded in the common VEPs characterized MA patients in comparison with MO and controls. Moreover, also in line with the TCD theory, the most cortical visual HFOs lacked habituation in both MO and MA [40]. The anatomical correlates of such defective thalamic control in MA are beginning to be understood [139–141, 146], and may be dynamically related to the distance from the last migraine attack [147].

## Conclusions

In summary, there are few neurophysiological features peculiar to the brain of patients with migraine with aura, such as frequent detection of an increase in amplitude to evoked potentials and peculiar abnormalities of functional connectivity at the EEG during resting-state. However, in most cases all the electrophysiological abnormalities – even those in common with MO – are more pronounced the more numerous and intense focal neurological symptoms characterize the aura. Intuitively we can say that most of the neurophysiological characteristics are certainly common to migraine patients with and without aura because most patients with migraine with aura also experience migraine attacks without aura [11]. On the other hand, pharmacological studies have shown that some drugs are able to stop the aura, but not the start of the migraine pain, clearly suggesting that the two phenomena are separate from the point of view of the underlying mechanisms [148]. The information coming from genetic studies is vaguer because at the moment no one has managed to demonstrate that genes certainly involved in the pathophysiology of familial hemiplegic migraine are also involved in the most common forms of migraine with and without aura [149]. Nevertheless, genome-wide association studies (GWAS) have shown that some genetic variants are associated with both migraine with and without aura, but they do not tell us whether they are associated with aura as such or with migraine pain that is in common [11]. Perfusion abnormalities that are likely to accompany migraine with aura, have also been detected in clinical cases of patients with migraine without aura, but during the pain phase and under intense visual stimulation, raising doubts about the possible auratic nature of the phenomenon [150].

Whatever the peculiar physiological characteristics of the migraineur with aura brain may be, taken alone it is not sufficient to explain all features of the migraine attack. In many patients some migraine-related symptoms may also be present during the intercritical period, and premonitory symptoms, associated with hypothalamic, brain stem and various cortical activations revealed on H_2_^15^O-PET scanning [151] may occur hours before aura and/or headache onset.

Supplementary studies are needed to clarify the exact relation between the electrocortical phenomena found outside the aura phase and during the aura itself. Studies correlating aura frequency and duration of the disorder with thalamic/thalamocortical activity in MA are necessary to test whether an abnormal cross-talk between the cortex and the thalamus – the latter area activated by CSD in animal models [152] –, could induce and/or worsen the interictal cortical abnormalities in MA. A better characterization of clinical/electrophysiological phenotypes of migraine with aura will allow the identification of selected migraine patients who may carry greater load of morpho-functional abnormalities, and may be hopefully the target for novel, tailored, therapeutic interventions. Finally, further studies combining functional neuroimaging and neurophysiological methods, simultaneously or deferred, in the same patient are desirable for the understanding of the exact anatomical correlates of the abnormal cerebral information processing related to migraine aura.

## Abbreviations

AEP: Auditory evoked potential
BAEP: Brainstem auditory evoked potentials
CSD: Cortical spreading depression
EEG: Electroencephalography
EP: Evoked potential
ERP: Event-related potential
FHM: Familiar hemiplegic migraine
HFO: High-frequency oscillation
MA: Migraine with aura
MEG: Magnetoelectroencephalography
MEP: Motor evoked potential
MO: Migraine without aura
nBR: Nociceptive blink reflex
PA: Persistent aura
PD: Photic driving
rTMS: repetitive transcranial magnetic stimulation
SFEMG: Single-fibre electromyography
SSEP: Somatosensory evoked potentials
SS-VEP: Steady-state visual evoked potential
TCD: Thalamo-cortical dysrhythmia
TMS: Transcranial magnetic stimulation
VEP: Visual evoked potential
